# Association of intensity-based walking steps and incident falls: a population-based cohort study

**DOI:** 10.7189/jogh.16.04382

**Published:** 2026-11-13

**Authors:** Ying Hu, Chuanchao Cao, Hongyi He, Yuqing Zhang, Chao Zeng, Guanghua Lei, Yilun Wang, Jie Wei

**Affiliations:** 1 Department of Orthopaedics, Xiangya Hospital, Central South University, Changsha, China; 2 Department of Epidemiology and Health Statistics, Xiangya School of Public Health, Central South University, Changsha, China; 3 Division of Rheumatology, Allergy, and Immunology, Department of Medicine, Massachusetts General Hospital, Harvard Medical School, Boston, Massachusetts, USA; 4 The Mongan Institute, Massachusetts General Hospital, Harvard Medical School, Boston, Massachusetts, USA; 5 Key Laboratory of Aging-related Bone and Joint Diseases Prevention and Treatment, Ministry of Education, Central South University, Changsha, China; 6 Hunan Key Laboratory of Joint Degeneration and Injury, Xiangya Hospital, Central South University, Changsha, China; 7 National Clinical Research Center for Geriatric Disorders, Xiangya Hospital, Central South University, Changsha, China; 8 Hunan Provincial Clinical Research Center for Joint Surgery, Xiangya Hospital, Central South University, Changsha, China

**Keywords:** walking, falls, hip fracture, cohort study

## Abstract

**Background:**

Falls are the leading cause of morbidity and mortality, yet they are largely preventable. While walking has well-established health benefits, its role in falls remains unclear. We aimed to examine the association between walking and fall risk.

**Methods:**

Walking steps were defined as purposeful walking steps (≥40 steps/min) and incidental walking steps (<40 steps/min), differentiated by walking intensity. We estimated hazard ratios (HRs) for incident falls using Cox proportional hazards models across the four walking step categories. Risk ratios (RRs) for the number of falls were estimated using Poisson regression. We also estimated the association between walking steps and the risk of incident hip fracture as the secondary outcome.

**Results:**

We included 94,422 participants aged 42–78 years from the UK Biobank, with a median follow-up of 6.87 years. We adjusted multivariable models for demographic, lifestyle, and clinical covariates. Compared with the lowest purposeful waking group (≤3999 steps/day), the second (HR = 0.75; 95% confidence interval (CI) = 0.60, 0.92), third (HR = 0.65; 95% CI = 0.52, 0.82), and fourth (HR = 0.68; 95% CI = 0.54, 0.86) group had lower risks of incident falls. Also, the RRs for the second (RR = 0.78; 95% CI = 0.68, 0.90), third (RR = 0.74; 95% CI = 0.64, 0.85), and fourth (RR = 0.72; 95% CI = 0.62, 0.84) group showed similar results. We also observed an inverse association between purposeful walking steps and hip fracture risk, while incidental walking steps showed no significant association.

**Conclusions:**

Higher purposeful walking steps were associated with a lower risk of incident falls, number of falls, and incident hip fracture, although residual confounding cannot be ruled out in this observational study. These findings may help inform future step-based recommendations for fall prevention.

Falls are among the most common and preventable causes of morbidity and mortality among older adults worldwide [1]. In the USA alone, over 14 million adults aged ≥65 years, experience falls each year, with 27.5% reporting at least one fall in the past 12 months [2]. Furthermore, age-adjusted rates of fatal falls increased 41% over the past ten years [3]. Falls-related mortality has more than doubled, with the crude mortality rate rising from 51.6 to 122.2 per 100,000 persons between 2000 and 2016 [4]. Among the non-fatal consequences of falls, 68% of individuals report a physical injury, and approximately 5–10% of all falls result in a fracture, with 98% of hip fractures attributable to falls [5–8]. Hip fractures, in particular, are the leading cause of disability and mortality among older adults [9]. As such, preventing falls in this population represents a major public health priority. Although researchers have identified numerous fall risk factors, including neurological conditions, frailty, auditory and visual impairments, and body mass index (BMI) [1,6,10], few are readily modifiable. Therefore, it is essential to identify risk factors that are both modifiable and amenable to intervention.

Walking is a widely accessible and commonly practised form of physical activity that confers various health benefits, including reduced risks of dementia and obesity, two known contributors to fall risk [11–13]. However, the relationship between walking and falls remains poorly understood. Previous studies suggest that walking intensity can be categorised into two distinct types: ‘purposeful walking’ and ‘incidental walking’, each characterised by different intensities [14]. Purposeful walking involves deliberate, coordinated, rhythmic movement at higher intensities, such as during exercise or commuting, and is typically sustained for longer durations [14,15]. In contrast, incidental walking encompasses spontaneous, unintentional movements performed at lower intensities [14]. However, prior studies examining walking steps and fall risk have primarily focused on total daily step counts or general physical activity measures [16], with inconsistent findings regarding their associations with falls [17]. Failing to account for walking intensity may obscure important nuances in the association between walking and fall risk. To date, the relationship between intensity-based walking steps and fall risk remains unclear. Therefore, distinguishing between purposeful and incidental walking steps, which reflect the accumulation of different walking types, may be crucial for developing step-based recommendations for fall prevention.

To address this knowledge gap, we aim to conduct prospective cohort studies using UK Biobank (UKB) data to examine the association between intensity-based daily step counts, specifically purposeful walking steps and incidental walking steps, and fall risk. Considering that fall-related hip fractures are among the leading causes of disability and mortality in older adults, we also examined the association between intensity-based walking step counts and the risk of incident hip fracture.

## METHODS

### Data source and study population

We used data from the UKB (application number 104208) and conducted this as a secondary analysis of the resource. Reporting of the study followed the GRABDROP guidance (Table S1 in the **Online Supplementary Document**) [18].

The UKB is a large prospective cohort comprising 502,357 participants aged 40–69 years, recruited between 2006 and 2010 [19]. All participants provided written informed consent. Baseline data were collected through questionnaires and physical assessments. A subset of 103,660 participants who consented to additional monitoring wore an Axivity AX3 accelerometer on their dominant wrist continuously for seven days. We excluded participants if they did not have valid accelerometer data at baseline, defined as wearing the device for at least 16 hours per day on a minimum of three days. We designated the date of baseline accelerometer data collection as the index date (*i.e.* start of follow-up). For the analysis of incident falls and incident hip fractures, we further excluded participants with a documented history of falls or hip fractures before the index date.

### Assessment of walking steps

We quantified walking steps and their intensity using the Verisene step-count algorithm. ‘Purposeful walking steps’ were steps accumulated at a cadence of ≥40 steps/min, whereas ‘incidental walking steps’ were the accumulation at <40 steps/min. We selected this cut-off based on previous studies showing that cadence can effectively characterise free-living stepping patterns, with higher cadences (≥40 steps/min) reflecting more sustained, purposeful ambulatory activity and lower cadences (<40 steps/min) reflecting predominantly incidental movement [12,14,20,21]. Daily purposeful and incidental walking steps were calculated for each participant by averaging the daily purposeful or incidental step counts on valid days, capturing both structured and spontaneous physical activity [11].

### Assessment of outcomes

The primary outcomes were incident falls and the number of falls during the follow-up period. We identified falls using ICD-10 codes W00-W19 in hospital inpatient records. We defined incident falls as the first fall diagnosis recorded after the index date among participants without a prior history of falls. We calculated the number of falls by counting all fall-related diagnoses from the index date to the end of follow-up (December 2021). The secondary outcome was an incident hip fracture, identified using ICD-10 codes S72.0–72.2. We defined incident hip fracture as the first recorded diagnosis after the index date among participants without a prior history of hip fracture.

### Assessment of other baseline characteristics

Baseline covariates were obtained from UKB data. These included age, sex, Townsend Deprivation Index, accelerometer wear time, BMI, education level, ethnicity, alcohol consumption, smoking status, vision, bone mineral density (BMD), history of fractured/broken bones, history of falls, osteoporosis, osteoarthritis, dementia, frailty, diabetes, daily sleep time, health status, serum vitamin D and calcium levels, and vitamin D and calcium supplementation [17,22–27] (Methods S1 in the **Online Supplementary Document**).

### Statistical analysis

To analyse incident falls, we first divided incident fall cases into four groups based on the quartile distribution of purposeful walking steps within the case population. We then selected integer values near these quartile thresholds to establish cut-off points for the entire study population. These cut-offs facilitated categorisation and improved interpretability for potential future clinical recommendations [28]. Specifically, participants were grouped according to purposeful walking steps as follows: reference group (≤3,999 steps/day), second group (4,000–5,999 steps/day), third group (6,000–7,999 steps/day), and fourth group (≥8,000 steps/day). To assess covariate balance across groups, we computed standardised mean differences, with values less than 0.1 indicating acceptable balance. We followed participants from the index date until the first fall diagnosis, loss to follow-up, death, or the end of the follow-up period (December 2021), whichever came first. We applied Fine–Gray proportional subdistribution hazards models, using age as the timescale, to examine the association between purposeful walking steps and incident falls, with death treated as a competing event [29]. Hazard ratios (HRs) and 95% confidence intervals (CIs) were calculated for each group, adjusting for age, sex, Townsend Deprivation Index, alcohol consumption status, accelerometer wear time, BMI, education level, ethnicity, vision, dementia, frailty, BMD, history of fractured/broken bones, osteoporosis, osteoarthritis, daily sleep time, and health status. We approximated the seven-year cumulative risk difference (RD) by multiplying the hazard differences estimated from the semiparametric additive hazards model by seven, assuming that the hazard difference remained approximately constant over follow-up. This approximation is reasonable given the low incidence of falls during the study period [30]. Missing data (<5%) were excluded from the analysis [31,32]. Model diagnostics included evaluating multicollinearity among covariates using variance inflation factors, assessing overall model fit with Cox–Snell residuals, and verifying the proportional hazards assumption with Schoenfeld residuals. To explore the dose-response relationship between purposeful walking steps and fall risk, we applied restricted cubic spline regression with four knots positioned at the medians of the four groups [33]. For the number of falls, we performed Poisson regression models to estimate relative risks (RRs) and 95% CIs, adjusting for the same covariates. We also used restricted cubic spline models to evaluate the dose-response relationship between purposeful walking steps and the number of falls. We also used a Cox proportional hazards model to examine the association between purposeful walking steps and the risk of incident fracture, adjusting for the same covariates. In this analysis, we excluded participants with a history of fracture.

We applied the same modelling approaches to analyse associations between incidental walking steps and the risk of incident falls, fall count, and incident hip fracture. We categorised participants into four groups based on incidental walking steps: reference group (≤2,999 steps/day), second group (3,000–3,999 steps/day), third group (4,000–4,999 steps/day), and fourth group (≥5,000 steps/day) [34].

To evaluate the independent effects of the two walking step categories, we also adjusted for incidental walking steps in models analysing purposeful walking steps, and *vice versa*. We also assessed the robustness of the findings by excluding participants with frailty or poor self-reported health at baseline. We also performed stratified analyses to examine whether associations varied by age, sex, BMI, and frailty status.

We used *R*, version 4.4.0 (R Core Team, Vienna, Austria) and SAS, version 9.4 (SAS Institute, Cary, North Carolina, USA) for all analyses, with a two-sided *P*-value <0.05 considered statistically significant (Supplementary Methods in the **Online Supplementary Document**).

## RESULTS

Among 98,248 UKB participants with available accelerometer data, 3826 were excluded due to invalid accelerometer recordings (**Table 1**). The final analytical sample included 94,422 individuals with a mean age of 61.79; 44.0% were men, and 90,932 reported no history of falls. Compared with participants in the reference group (≤3,999 steps/day), those in higher purposeful walking step groups were generally younger and had lower BMI, lower prevalence of frailty and osteoarthritis, better vision and self-reported health status, higher educational attainment, longer average sleep duration, and higher daily incidental walking steps.

**Table 1:** Baseline characteristics of purposeful walking steps categories for incident falls*

		Purposeful walking steps				
	**Overall**	**Reference (≤3,999)**	**Second (4,000–5,999)**	**Third (6,000–7,999)**	**Fourth (≥8,000)**	**SMD**
**Participants**	90,932	11,813	24,836	26,786	27,497	
**Age (years), x̄ (SD)**	61.79 (7.85)	64.74 (7.41)	62.52 (7.87)	61.25 (7.83)	60.38 (7.62)	0.31
**Men**	4,0012 (44.0)	5,800 (49.1)	11,028 (44.4)	11,486 (42.9)	11,698 (42.5)	0.07
**White**	87,783 (96.5)	11,419 (96.7)	23,961 (96.5)	25,873 (96.6)	26,530 (96.5)	0.01
**BMI (kg/m^2^), x̄ (SD)**	26.69 (4.5)	29.26 (5.7)	27.27 (4.5)	26.23 (4.1)	25.52 (3.8)	0.44
**TDI, x̄ (SD)**	−1.74 (2.8)	−1.62 (2.9)	−1.87 (2.7)	−1.82 (2.8)	−1.58 (2.9)	0.07
**College**	39,192 (43.5)	4,137 (35.6)	10,120 (41.2)	12,067 (45.5)	12,868 (47.2)	0.13
**Alcohol**						0.07
Current	85,767 (94.4)	10,850 (91.9)	23,373 (94.2)	25,449 (95.1)	26,095 (95.0)	
Never	2,627 (2.9)	482 (4.1)	762 (3.1)	674 (2.5)	709 (2.6)	
Previous	2,458 (2.7)	468 (4.0)	681 (2.7)	645 (2.4)	664 (2.4)	
**Vision problem**	6,101 (18.8)	1,181 (27.4)	1,804 (20.4)	1,527 (16.0)	1,589 (16.3)	0.16
**BMD (g/cm^2^), x̄ (SD)**	0.55 (0.1)	0.54 (0.1)	0.55 (0.1)	0.55 (0.1)	0.56 (0.1)	0.05
**History of fractured/broken bones**	7,216 (7.9)	933 (7.9)	1,914 (7.7)	2,109 (7.9)	2,260 (8.2)	0.02
**Osteoporosis**	883 (1.0)	218 (1.8)	218 (0.9)	216 (0.8)	231 (0.8)	0.05
**Dementia**	31 (0.0)	11 (0.1)	8 (0.0)	8 (0.0)	4 (0.0)	0.02
**Osteoarthritis**	7,216 (7.9)	1,635 (13.8)	2,174 (8.8)	1,836 (6.9)	1,571 (5.7)	0.15
**Frailty**	1,654 (1.8)	732 (6.2)	523 (2.1)	243 (0.9)	156 (0.6)	0.18
**Health status**						0.33
Excellent	19,850 (21.9)	1,412 (12.0)	4,781 (19.3)	6,335 (23.7)	7,322 (26.7)	
Good	54,534 (60.1)	6,364 (54.1)	15,084 (60.9)	16,358 (61.2)	16,728 (60.9)	
Fair	14,077 (15.5)	3,095 (26.3)	4,238 (17.1)	3,652 (13.7)	3,092 (11.3)	
Poor	2,250 (2.5)	897 (7.6)	653 (2.6)	391 (1.5)	309 (1.1)	
**Total sleep time (hours/d), x̄ (SD)**	8.42 (1.4)	8.92 (1.7)	8.57 (1.4)	8.37 (1.3)	8.13 (1.3)	0.29
**Accelerometer wear time (hours/d), x̄ (SD)**	6.68 (0.6)	6.67 (0.6)	6.69 (0.6)	6.69 (0.6)	6.66 (0.7)	0.03
**Incidental walking steps (steps/d)**						1.09
Reference (≤2,999)	16,465 (18.1)	6,973 (59.0)	5,051 (20.3)	2,625 (9.8)	1,816 (6.6)	
Second (3,000–3,999)	31,691 (34.9)	4,190 (35.5)	11,802 (47.5)	9,120 (34.0)	6,579 (23.9)	
Third (4,000–4,999)	27,706 (30.5)	627 (5.3)	6,882 (27.7)	10,520 (39.3)	9,677 (35.2)	
Fourth (≥5,000)	15,070 (16.6)	23 (0.2)	1,101 (4.4)	4,521 (16.9)	9,425 (34.3)	

BMI – body mass index, BMD – bone mineral density, SD – standard deviation, SMD – standardised mean difference, TDI – Townsend Deprivation Index, x̄ – mean

*Variables are presented as n (%) unless specified otherwise.

Over a mean follow-up of seven years, 2985 participants experienced incident falls. For purposeful walking steps, fall incidence rates were highest in the reference group (7.93), followed by the second (4.97), third (4.22), and fourth (3.86) per 1,000 person-years. Compared with the reference group, the second (HR = 0.75; 95% CI = 0.60, 0.92), third (HR = 0.65; 95% CI = 0.52, 0.82), and fourth (HR = 0.68; 95% CI = 0.54, 0.86) groups had lower fall risk. Given the moderate positive correlation between purposeful and incidental walking steps (*r =* 0.53; *P* < 0.001), the association between purposeful walking steps and incident falls remained significant after adjusting for incidental walking steps (**Table 2**). Restricted cubic spline analysis revealed an inverse association, with HRs for incident falls decreasing significantly as purposeful walking steps increased (**Figure 1**, Panel A). The seven-year cumulative RDs decreased for the second (RD = −1.46; 95% CI = −2.54, −0.38), third (RD = −1.83; 95% CI = −2.93, −0.74), and fourth (RD = −1.70; 95% CI = −2.84, −0.58) groups. E-values were 1.85 in the second, 2.17 in the third, and 2.00 in the fourth group, indicating that potential residual confounders must have an association of at least 1.85 with both purposeful walking steps and incident falls to nullify the inverse association. Subgroup analyses showed inverse associations between purposeful walking steps and incident falls among male participants aged >60 years who were non-frail and non-obese, whereas these associations were not statistically significant in the corresponding comparison subgroups (Figure S3 in the **Online Supplementary Document**). There was no evidence of substantial multicollinearity (all variance inflation factor values <5). The model showed a good fit (Figure S2 in the **Online Supplementary Document**) and met the proportional hazards assumption (all *P* > 0.05).

**Table 2:** Association between walking steps and the risk of incident falls

						HR (95% CI)*			
	**n**	**Incident case (incident rate)**	**x̄ follow-up time (years)**	**Rate difference (95% CI)**	**% RD (95% CI)**	**Crude model**	**Model 1**	**Model 2**	**Model 3**
**Purposeful walking steps**									
Reference (≤3,999)	11,813	623 (7.93)	6.65	ref	ref	ref	ref	ref	ref
Second (4,000–5,999)	24,836	845 (4.97)	6.85	−2.96 (−3.67, −2.25)	−1.46 (−2.54, −0.38)	0.73 (0.65, 0.80)	0.75 (0.60, 0.92)	0.79 (0.63, 0.99)	0.82 (0.64, 1.05)
Third (6,000–7,999)	26,786	781 (4.22)	6.91	−3.71 (−4.40, −3.02)	−1.83 (−2.93, −0.74)	0.67 (0.60, 0.75)	0.65 (0.52, 0.82)	0.71 (0.56, 0.91)	0.71 (0.55, 0.93)
Fourth (≥8,000)	27,497	736 (3.86)	6.94	−4.07 (−4.75, −3.39)	−1.70 (−2.84, −0.58)	0.65 (0.59, 0.73)	0.68 (0.54, 0.86)	0.75 (0.58, 0.98)	0.74 (0.56, 0.98)
**Incidental walking steps**									
Reference (≤2,999)	16,465	695 (6.27)	6.73	ref	ref	ref	ref	ref	ref
Second (3,000–3,999)	31,691	1002 (4.60)	6.87	−1.67 (−2.22, −1.12)	−0.47 (−1.29, 0.35)	0.81 (0.74, 0.90)	0.81 (0.66, 0.98)	0.88 (0.71, 1.08)	0.87 (0.70, 1.09)
Third (4,000–4,999)	27,706	846 (4.42)	6.91	−1.85 (−2.40, −1.30)	−0.65 (−1.53, 0.22)	0.83 (0.75, 0.92)	0.73 (0.59, 0.91)	0.83 (0.66, 1.06)	0.87 (0.68, 1.11)
Fourth (≥5,000)	15,070	442 (4.23)	6.93	−2.04 (−2.65, −1.43)	−0.78 (−1.80, 0.23)	0.85 (0.75, 0.96)	0.69 (0.53, 0.90)	0.79 (0.59, 1.07)	0.82 (0.61, 1.12)

CI – confidence interval, HR – hazard ratio, RD – risk difference, ref – reference, x̄ – mean

*Model 1 is adjusted for age, sex, deprivation, alcohol consumption status, accelerometer wear time, BMI, education level, ethnicity, vision status, total sleep time, frailty, BMD, dementia, history of fractured/broken bones, osteoporosis, osteoarthritis, and health status; Model 2 is adjusted for all Model 1 variables plus purposeful walking steps in the incidental walking analyses and incidental walking steps in the purposeful walking analyses; Model 3 excludes participants with frailty and poor health status and is adjusted for Model 2.

**Figure 1:**
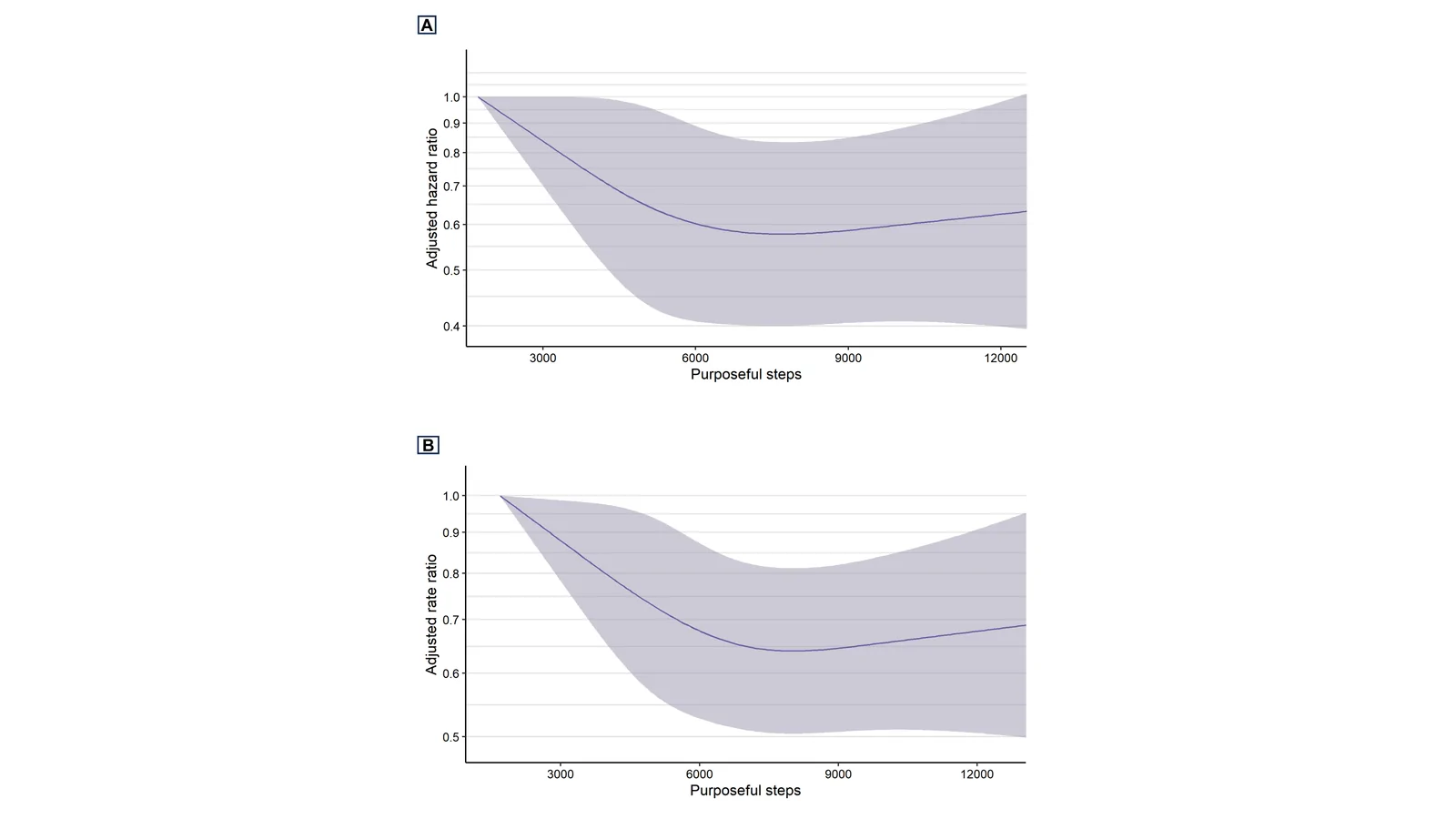
Dose-response relationship between purposeful walking steps and the risk of incident and number of falls. **Panel A.** Purposeful walking steps and incident falls. **Panel B.** Purposeful walking steps and number of falls. Hazard ratios and risk ratios were adjusted for multiple covariates, including age, sex, deprivation, alcohol consumption status, accelerometer wear time, BMI, education level, ethnicity, vision status, total sleep time, frailty, BMD, dementia, history of fractured/broken bones, osteoporosis, osteoarthritis, health status, and incidental walking steps. BMI – body mass index, BMD – bone mineral density.

Purposeful walking steps were also inversely associated with the number of falls in the second (RR = 0.78; 95% CI = 0.68, 0.90), third (RR = 0.74; 95% CI = 0.64, 0.85), and fourth (RR = 0.72; 95% CI = 0.62, 0.84) group (**Table 3**). The association remained robust after adjusting for incidental steps. Restricted cubic spline regression revealed a curvilinear relationship, with a pronounced reduction in fall risk at moderate levels of purposeful walking, which attenuated at higher step volumes (**Figure 1**, Panel B). E-values were 1.77 in the second, 1.92 in the third, and 1.96 in the fourth group. Sensitivity analyses excluding participants with frailty or poor health status yielded consistent results.

**Table 3:** Association between walking steps and number of falls

			RR (95% CI)*			
	**n**	**Number of falls (%)**	**Crude model**	**Model 1**	**Model 2**	**Model 3**
**Purposeful walking steps**						
Reference (≤3,999)	12,468	1,529 (12.26)	ref	ref	ref	ref
Second (4,000–5,999)	25,830	2,053 (7.95)	0.65 (0.61, 0.69)	0.78 (0.68, 0.90)	0.81 (0.70, 0.94)	0.82 (0.70, 0.96)
Third (6,000–7,999)	27,686	1,862 (6.72)	0.55 (0.51, 0.59)	0.74 (0.64, 0.85)	0.77 (0.66, 0.90)	0.77 (0.65, 0.91)
Fourth (≥8,000)	28,438	1,849 (6.50)	0.53 (0.50, 0.57)	0.72 (0.62, 0.84)	0.76 (0.64, 0.90)	0.73 (0.61, 0.88)
**Incidental walking steps**						
Reference (≤2,999)	17,208	1,681 (9.77)	ref	ref	ref	ref
Second (3,000–3,999)	32,857	2,404 (7.32)	0.75 (0.70, 0.80)	0.84 (0.74, 0.96)	0.90 (0.79, 1.03)	0.90 (0.78, 1.04)
Third (4,000–4,999)	28,722	2,085 (7.26)	0.74 (0.70, 0.79)	0.82 (0.71, 0.94)	0.91 (0.78, 1.06)	0.94 (0.80, 1.10)
Fourth (≥5,000)	15,635	1,123 (7.18)	0.74 (0.68, 0.79)	0.77 (0.65, 0.91)	0.88 (0.73, 1.05)	0.91 (0.75, 1.11)

CI – confidence interval, RR – risk ratio, ref – reference

*Model 1 is adjusted for age, sex, deprivation, alcohol consumption status, accelerometer wear time, BMI, education level, ethnicity, vision status, total sleep time, frailty, BMD, dementia, history of fractured/broken bones, osteoporosis, osteoarthritis, and health status; Model 2 is adjusted for all Model 1 variables plus purposeful walking steps in the incidental walking analyses and incidental walking steps in the purposeful walking analyses; Model 3 excludes participants with frailty and poor health status and is adjusted for Model 2.

Compared with those in the reference group (≤2,999 steps/day), participants in the higher incidental walking step groups were more likely to be female, younger, have lower BMI, better vision and general health, shorter sleep duration, and higher purposeful walking steps (Table S2 in the **Online Supplementary Document**). We initially observed an inverse association between incidental walking steps and the risk of incident falls; however, after further adjustment for purposeful walking steps, this association became non-significant (**Table 2**). We observed a similar pattern for the number of falls (**Table 3**). Restricted cubic spline regression revealed a modest inverse association of incidental walking steps with either incident falls or the number of falls, but statistical significance was reached only at moderate step levels (**Figure 2**, Panel A and B). In sensitivity analyses, incidental walking steps were not significantly associated with either incident or the number of falls.

**Figure 2:**
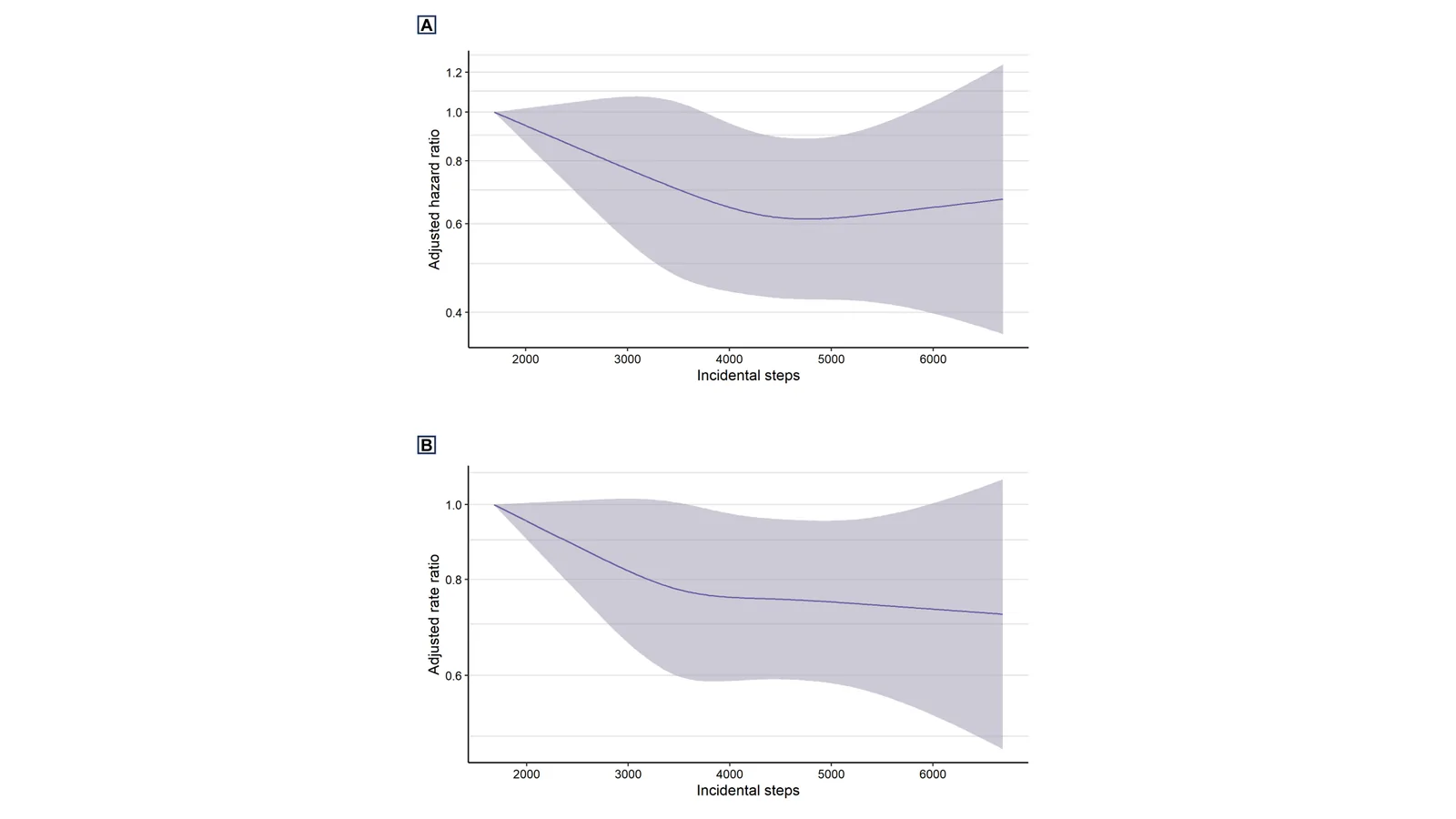
Dose-response relationship between incidental walking steps and the risk of incident and number of falls. **Panel A.** Incidental walking steps and incident falls. **Panel B.** Incidental walking steps and number of falls. Hazard ratios and risk ratios were adjusted for multiple covariates, including age, sex, deprivation, alcohol consumption status, accelerometer wear time, BMI, education level, ethnicity, vision status, total sleep time, frailty, BMD, dementia, history of fractured/broken bones, osteoporosis, osteoarthritis, health status, and purposeful walking steps. BMI – body mass index, BMD – bone mineral density.

We also reported baseline characteristics stratified by purposeful and incidental steps for the analysis of incident hip fracture. Participants with higher purposeful or incidental walking steps tended to be younger, have lower BMI, better self-reported health, shorter sleep duration, and higher levels of the other type of walking. Participants in higher purposeful walking were more likely to have higher educational attainment, while participants in higher incidental walking groups were more likely to be female. (Table S3 and S4 in the **Online Supplementary Document**). Purposeful walking steps were inversely associated with hip fracture risk. Compared with the reference group, the second (HR = 0.63; 95% CI = 0.39, 1.02), third (HR = 0.49; 95% CI = 0.29, 0.83), and fourth (HR = 0.36; 95% CI = 0.21, 0.62) group had lower risks of incident hip fracture (Table S5 in the **Online Supplementary Document**). Restricted cubic spline regression revealed a dose-dependent inverse trend between purposeful walking and hip fracture risk (Figure S1 in the **Online Supplementary Document**). However, we found no significant association between incidental walking steps and hip fracture risk in either the Cox proportional hazards model or the restricted cubic spline regression.

## DISCUSSION

In this prospective population-based cohort study, higher-intensity walking, measured as purposeful walking steps, was significantly associated with lower risks of incident falls, falls, and hip fracture. In contrast, lower-intensity walking, characterised as incidental walking steps, showed no significant association with these outcomes. These findings suggest that accumulating more high-intensity walking may confer greater protection against falls and related injuries in the general population [35].

### Compared with previous studies

To date, few studies have examined the association between walking steps and incident falls and consequent events. Lloyd *et al.* reported that individuals who walked more slowly or accumulated fewer steps were more likely to experience injurious falls [17]. However, their analysis did not consider walking intensity, despite previous evidence indicating that walking intensity is significantly associated with falls [36]. Distinct walking intensities may reflect different movement patterns; relying solely on step counts without considering intensity may lead to misleading conclusions. For instance, one study found that self-reported brisk walking was associated with lower odds of non-injurious falls than no physical activity [37], whereas another reported higher fall incidence with higher total step counts [38]. These inconsistent findings may reflect methodological differences or selection bias, and neither study identified specific identity-based walking patterns that could inform fall prevention strategies. To address this gap, we distinguished between purposeful and incidental walking steps within total daily step counts, allowing us to more precisely identify walking patterns associated with fall risk. Mutual adjustment enabled us to examine the independent associations of purposeful and incidental walking steps with fall risk. Purposeful walking steps remained significantly associated with incident fall risk, whereas the association for incidental walking steps was non-significant. These findings suggest that the association for incidental walking may partially overlap with purposeful walking, whereas purposeful walking showed a more robust independent association with fall risk. Our findings on hip fracture are also broadly consistent with previous studies reporting inverse associations between moderate physical activity and hip fracture risk [39]. However, these studies did not specifically examine the association between walking steps and hip fracture risk, although walking steps represent an objective, readily measurable, and clinically meaningful indicator of physical activity.

### Potential explanations

Several mechanisms may explain the association between higher purposeful walking steps and reduced fall and hip fracture risk. First, previous studies show that exercise interventions, particularly those targeting balance and lower-extremity strength, can effectively reduce fall risk among individuals at average risk [6]. Walking, as a primary form of bipedal locomotion, is closely associated with lower body muscle strength [40,41]. Specifically, purposeful walking, characterised by sustained, rhythmic movements at higher cadences, likely reflects deliberate physical activity, whereas incidental walking typically involves low-effort, sporadic movements, such as standing stretches or short indoor walks. As such, purposeful walking may enhance lower-body muscle power and neuromuscular coordination, both critical components of fall prevention. The observed association with hip fracture risk may be partly explained by the lower risk of falls, as falls are a major cause of hip fractures in older adults [42]. Additionally, previous studies have suggested that regular physical activity may help maintain BMD and musculoskeletal health, which could further reduce fracture susceptibility. Together, these findings support the potential relevance of purposeful walking to both fall and fracture prevention. Second, low levels of purposeful walking may indicate low overall physical activity, which could result in decreased outdoor exposure and, consequently, reduced vitamin D synthesis. Vitamin D deficiency has been associated with impaired muscle function and an elevated risk of falls [43,44], further supporting the protective role of higher-intensity walking.

### Strengths and limitations

Several strengths of our study are worth noting. First, we used accelerometers to objectively and prospectively measure both walking steps and intensity. This approach provides more reliable and precise data than self-reported measures, which are susceptible to recall bias. Second, by concurrently assessing both step quantity and intensity, we could differentiate specific walking patterns, particularly higher-intensity walking (*i.e.* purposeful walking), that were associated with lower fall risk. Third, the consistent inverse association between purposeful walking steps and incident falls, number of falls, and risk of incident hip fracture enhances the robustness and generalisability of the primary findings.

This study also has several limitations. First, we assessed walking behaviour only once at baseline over a seven-day period, which may not fully capture long-term habitual walking patterns. However, walking is generally considered a stable behaviour over time [21]. Previous studies have shown moderate-to-good reproducibility of accelerometer-derived step measures at the population level, including repeated assessments among UKB participants [45]. Second, the use of an accelerometer may have introduced reactivity bias, whereby participants increased their activity levels while being monitored, potentially leading to an overestimation of average step counts compared with the general population or those who declined to participate in the accelerometer sub-study [46]. Finally, as with all observational studies, residual confounding and reverse causation cannot be completely ruled out.

### Clinical implications

Recent guidelines recommend engaging in physical activity as a strategy to reduce fall risk [47]. Previous studies have explored the associations between different intensities of physical activity and fall outcomes; the specific types of physical activity that are most effective remain unclear [38]. Although walking has been suggested to lower fall incidence and recurrence, conflicting findings have raised concerns that daily walking may, in some cases, increase fall risk. These inconsistencies highlight the importance of clarifying the role of walking intensity, an arguably more informative metric of physical activity than overall moderate-to-vigorous physical activity, in guiding fall prevention efforts [48].

Our study highlights the importance of distinguishing walking steps by intensity when developing fall prevention recommendations. Although the incidental walking steps (*i.e.* low-intensity) were not significantly associated with fall risk, this should not be interpreted as evidence that walking is ineffective. Rather, it highlights that step count alone is an insufficient metric without accounting for intensity. Furthermore, associations between purposeful walking steps and fall risk may vary by baseline characteristics, including frailty status and demographic or clinical factors such as age, sex, and BMI. These findings highlight the importance of cautious, individualised interpretation when translating these observational results into fall-prevention recommendations.

These findings offer practical insights for public health strategies, emphasising the value of increasing high-intensity walking to prevent falls. Notably, a previous population-based study found that swimming was the only activity associated with a lower fall rate among men [49]. Our results extend this literature by identifying purposeful walking, an accessible, intensity-driven form of physical activity, as a potentially effective fall prevention strategy. Given the established benefits of functional exercise in enhancing balance and lower-extremity strength, purposeful walking may be a pragmatic, scalable prevention strategy to reduce fall risk. Furthermore, previous studies have reported inverse associations between physical activity and fracture risk, although the optimal type and intensity of activity for fracture prevention remain uncertain. Our findings provide additional evidence that purposeful walking may inform future strategies to prevent fall-related hip fracture in ageing populations.

Future studies incorporating repeated accelerometer measurements and interventional designs are warranted to further clarify the long-term associations between purposeful walking steps, falls, and fracture prevention.

## CONCLUSIONS

More purposeful walking steps per day were associated with lower risks of incident falls, fewer falls, and hip fracture. Our findings provide important insights into the role of walking intensity in fall prevention and support integrating intensity-based walking recommendations into public health strategies to reduce the burden of falls and fall-related injuries.

## Additional material


Online Supplementary Document

## Data Availability

**Data availability:** The data used in this study can be accessed upon application. Requests for data access should be made directly to the UKB.
