# Supplementary material for: Association of intensity-based walking steps and incident falls: a population-based cohort study
Source: J Glob Health. 2026 Nov 13;16:04382. doi: 10.7189/jogh.16.04382 (PMC13761200; doi:10.7189/jogh.16.04382)
Supplement: Supplementary file 1 [file jogh-16-04382-s001.pdf]

Supplement to: Hu Y, Cao C, He H, Zhang Y, Zeng C, Lei G, Wang Y, Wei J. Association of intensity-based walking steps and incident falls: a population-based cohort study. J Glob Health. 2026;16:04382.

Content

Supplementary Methods 2

Figure S1 Dose-response relationship between purposeful walking steps, incidental walking steps, and the risk of incident hip fracture. 3

Figure S2 Assessment of overall model fit using Cox-Snell residuals. 5

Figure S3 Forest plot of subgroup analyses for the association between purposeful walking steps and incident falls 6

Table S1. Adherence to JoGH's GRABDROP guidelines items. 7

Table S2 Baseline characteristics of incidental walking steps categories for incident falls. 9

Table S3 Baseline characteristics of purposeful walking steps categories for incident hip fracture........ 11

Table S4 Baseline characteristics of incidental walking steps categories for incident hip fracture........ 13

Table S5 Association between the walking steps and the risk of incident hip fracture. 15

References 16

Supplementary Methods

Assessment of other baseline characteristics

Age, sex, and Townsend deprivation index were acquired from the local National Health Service (NHS), while accelerometer wear time data and daily sleep time were derived from accelerometer data. BMI was calculated from manually measured height and weight. Education level, ethnicity, alcohol consumption, smoking status, health status, history of fractured/broken bones, diabetes, vitamin D and calcium supplementation, and vision were assessed via a touchscreen questionnaire. Serum vitamin D and calcium levels were measured using CLIA analysis (DiaSorin Ltd. LIASON XL) and Arsenazo III analysis (Beckman Coulter AU5800), respectively. BMD was estimated via the Quantitative Ultrasound Index measured at the calcaneus. OA, OP, and dementia were identified based on ICD-10 codes. Frailty status was assessed using the five-item criteria described by Hanlon et al. Participants meeting three or more criteria were classified as frail.[1,2]

R package used in the analysis

The main R packages used included “survival”, “cmprsk”, “rms”, and “car” packages.

A

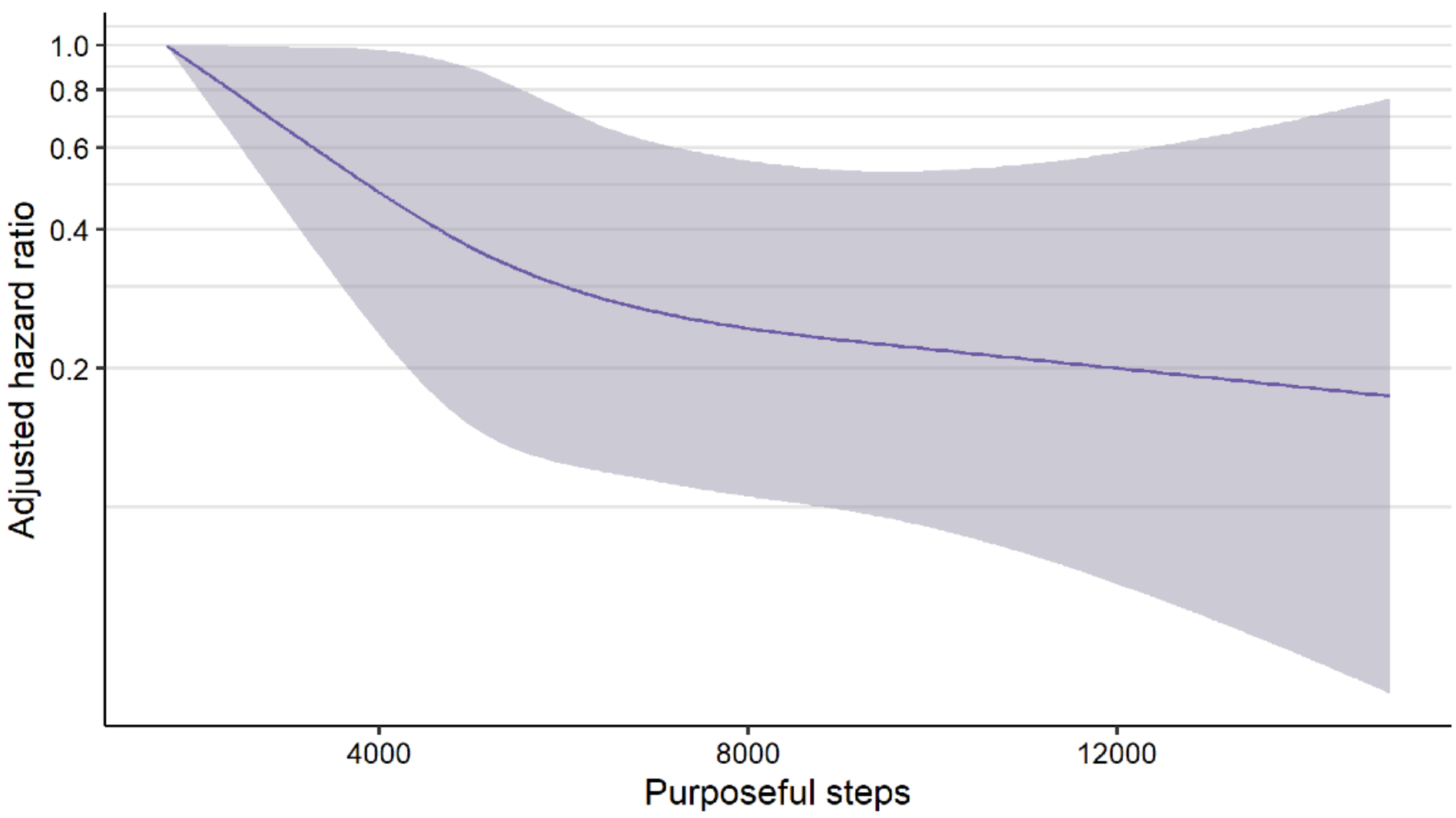

B

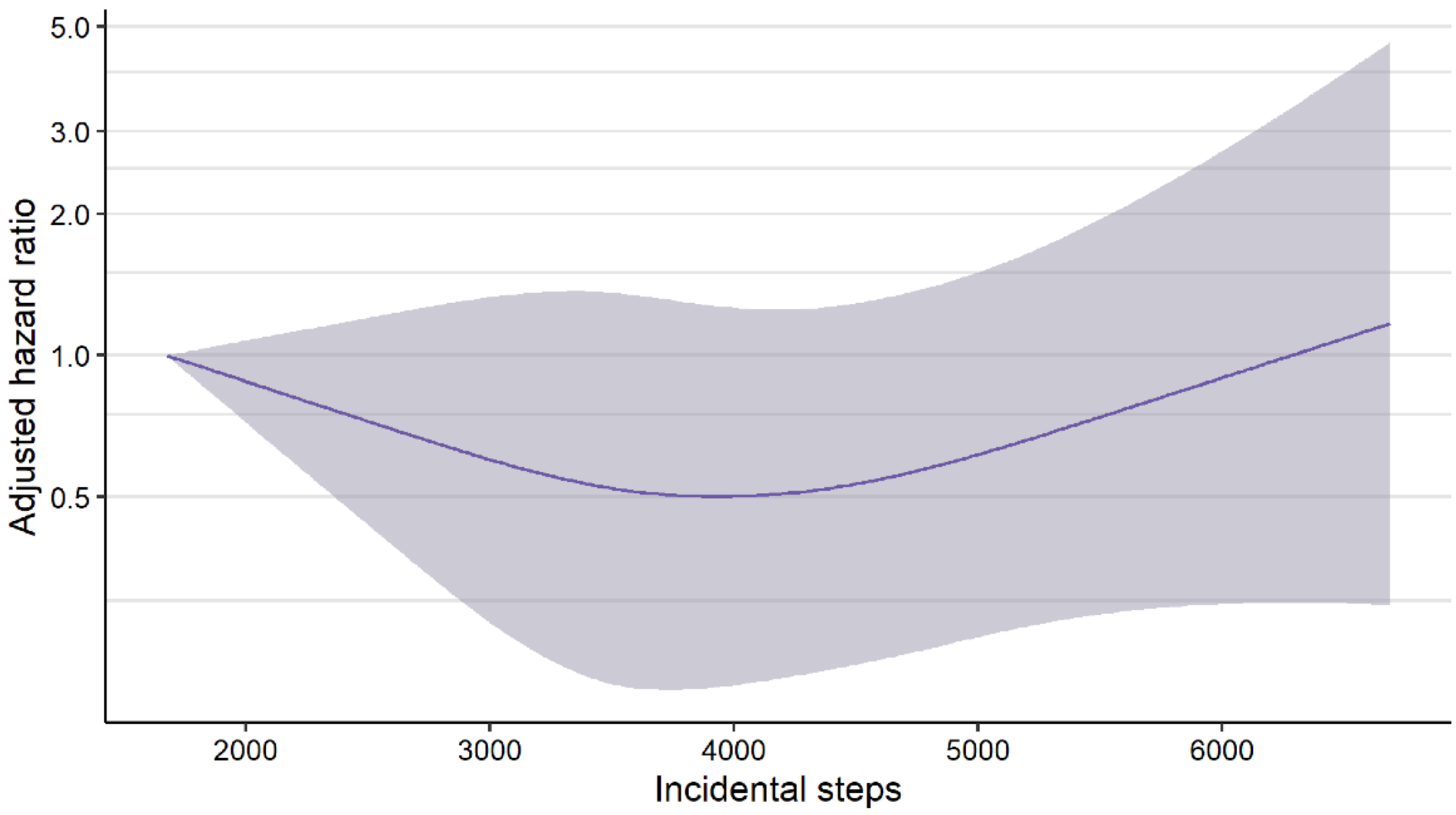

Figure S1 Dose-response relationship between purposeful walking steps, incidental walking steps, and the risk of incident hip fracture.

Panel A. Purposeful walking steps and incident hip fracture. **Panel B.** Incidental walking steps and hip fracture. The association between purposeful walking steps, incidental walking steps, and the risk of incident hip fracture was analyzed using restricted cubic spline regression models, with four knots placed at the median of the four groups of categorical exposures. The y-axis is a log scale with the shaded area representing 95% confidence intervals. Hazard ratios were adjusted for age, sex, ethnicity, education level, deprivation, alcohol consumption status, smoking status, health status, accelerometer wear time, BMI, daily sleep time, frailty, BMD, osteoporosis, osteoarthritis, diabetes, history of falls, serum vitamin D level, serum calcium level, vitamin D supplementation, and calcium supplementation,

with mutual adjustment between purposeful and incidental walking steps in the models. BMI – body mass index; BMD – bone mineral density.

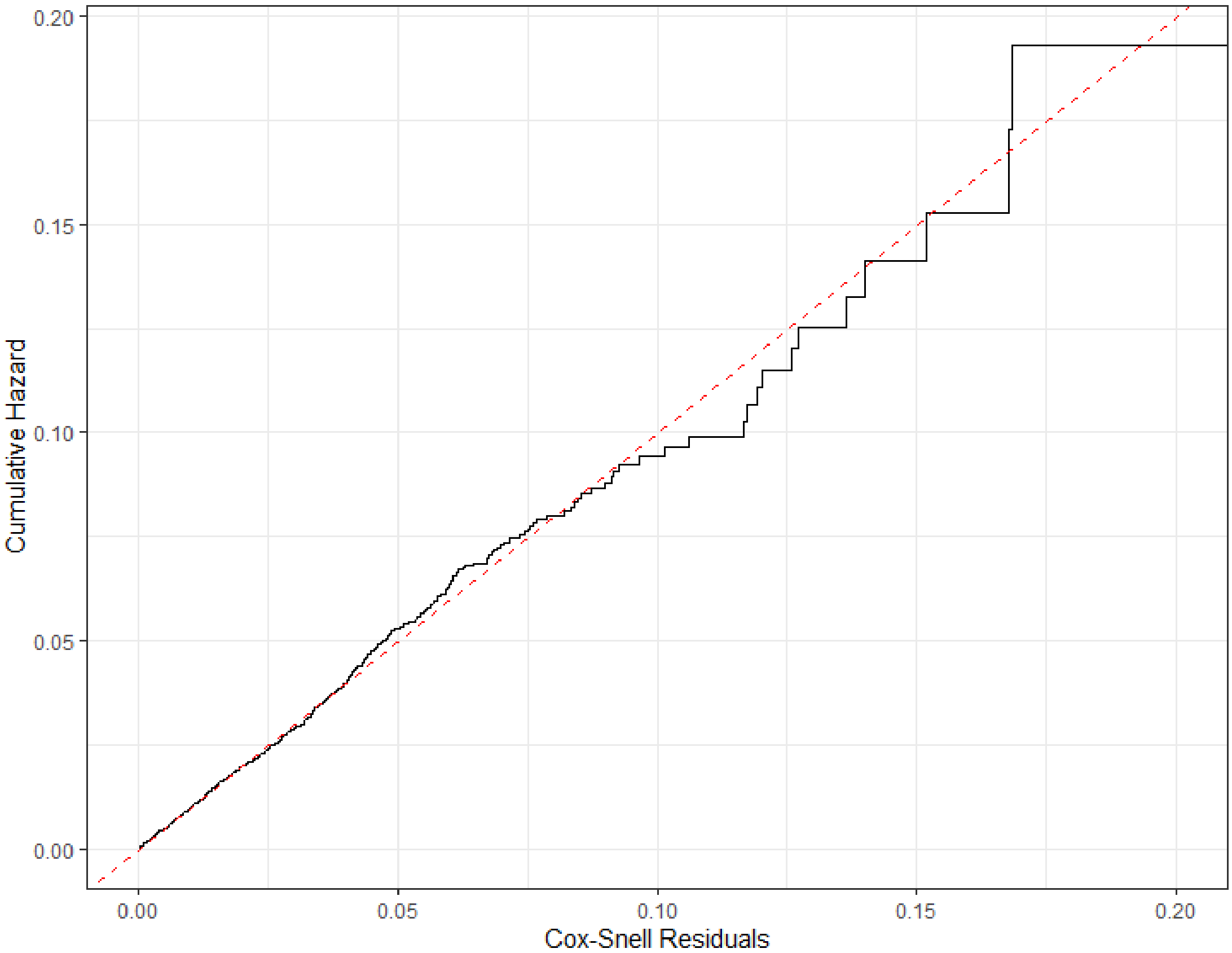

Figure S2 Assessment of overall model fit using Cox-Snell residuals.

The fully adjusted Cox proportional hazards model was adjusted for age, sex, deprivation, alcohol consumption status, accelerometer wear time, BMI, education level, ethnicity, vision status, daily sleep time, frailty, BMD, dementia, history of fractured/broken bones, osteoporosis, osteoarthritis, health status, and incidental walking steps. The Nelson–Aalen cumulative hazard estimate of the Cox–Snell residuals was plotted against the residuals themselves. BMI – body mass index; BMD – bone mineral density.

Subgroup	N	Cases	HR (95%CI)
**< 60 years**			
Reference	5191	152	1.00 (reference)
Second	13827	314	0.91 (0.59, 1.41)
Third	16823	348	0.74 (0.47, 1.17)
Fourth	18807	370	0.78 (0.49, 1.23)
**≥ 60 years**			
Reference	6622	471	1.00 (reference)
Second	11009	531	0.69 (0.54, 0.88)
Third	9963	433	0.63 (0.48, 0.82)
Fourth	8690	366	0.64 (0.48, 0.85)
**Male**			
Reference	5800	292	1.00 (reference)
Second	11028	312	0.73 (0.52, 1.02)
Third	11486	264	0.65 (0.45, 0.93)
Fourth	11698	231	0.51 (0.35, 0.76)
**Female**			
Reference	6013	331	1.00 (reference)
Second	13808	533	0.78 (0.59, 1.03)
Third	15300	517	0.68 (0.51, 0.92)
Fourth	15799	505	0.79 (0.58, 1.07)
**Non-obesity**			
Reference	7316	377	1.00 (reference)
Second	19034	649	0.72 (0.56, 0.93)
Third	22532	654	0.61 (0.47, 0.79)
Fourth	24365	652	0.62 (0.47, 0.80)
**Obesity**			
Reference	4430	237	1.00 (reference)
Second	5750	193	0.76 (0.51, 1.14)
Third	4218	125	0.80 (0.49, 1.30)
Fourth	3101	79	0.98 (0.59, 1.64)
**Non-frailty**			
Reference	11081	560	1.00 (reference)
Second	24313	821	0.77 (0.62, 0.96)
Third	26543	769	0.66 (0.52, 0.83)
Fourth	27341	730	0.68 (0.54, 0.87)
**Frailty**			
Reference	732	63	1.00 (reference)
Second	523	24	0.24 (0.07, 0.80)
Third	243	12	0.78 (0.19, 3.18)
Fourth	156	6	1.42 (0.43, 4.72)

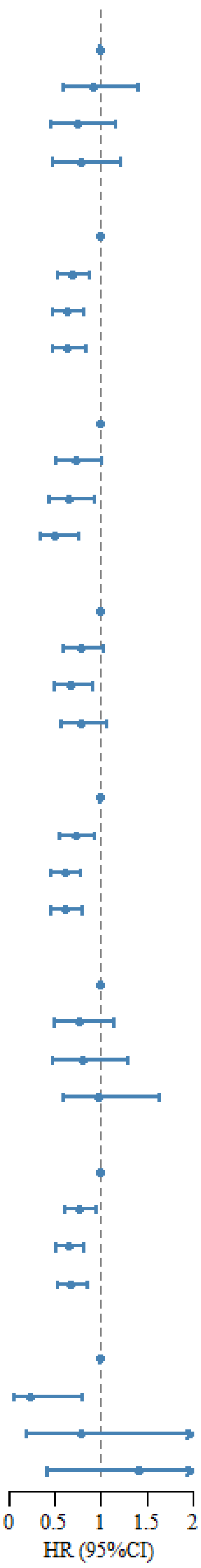

Figure S3 Forest plot of subgroup analyses for the association between purposeful walking steps and incident falls

Associations between purposeful walking steps and incident falls were assessed across subgroups using Fine–Gray proportional subdistribution hazards models. HRs were adjusted for age, sex, deprivation, alcohol consumption status, accelerometer wear time, BMI, education level, ethnicity, vision status, total sleep time, frailty, BMD, dementia, history of fractured/broken bones, osteoporosis, osteoarthritis, and health status. HR – hazard ratio; CI – confidence interval.

Table S1. Adherence to JoGH's GRABDROP guidelines items.

**JoGH guideline items**
1. Please list all papers published by each co-author in previous 3 years that were based on secondary analysis of a big data repository
(1) Yilun Wang; Yuqing Zhang; Chao Zeng; Guanghua Lei; Jie Wei. Target Serum Urate Achievement and Chronic Kidney Disease Progression in Patients With Gout and Kidney Disease [JAMA Intern Med 2025 Jan]<br>(2) Yuqing Zhang; Chao Zeng; Guanghua Lei; Jie Wei. Global burden of early-onset osteoarthritis, 1990-2019: results from the Global Burden of Disease Study 2019. [Ann Rheum Dis 2024 Jun]<br>(3) Hongyi He; Yilun Wang; Yuqing Zhang; Chao Zeng; Guanghua Lei; Jie Wei. Association of walking with incident knee osteoarthritis: a prospective cohort study using data from the UK Biobank. [Ann Rheum Dis 2025 Apr]<br>(4) Jie Wei; Chao Zeng; Guanghua Lei; Yuqing Zhang. Lowering serum urate with urate-lowering therapy to target and incident fracture among people with gout. [Arthritis Rheumatol 2023 Aug]<br>(5) Jie Wei; Chao Zeng; Guanghua Lei; Yuqing Zhang. Weight Loss Induced by Antiobesity Medications and All-Cause Mortality Among Patients With Knee or Hip Osteoarthritis. [Arthritis Rheumatol 2024 Apr]<br>(6) Jie Wei; Yilun Wang; Chao Zeng; Guanghua Lei; Yuqing Zhang. Weight Loss After Receiving Anti-Obesity Medications and Gout Among Individuals With Overweight and Obese: A Population-Based Cohort Study [Arthritis Rheumatol 2025 Mar]<br>(7) Jie Wei; Yilun Wang; Chao Zeng; Guanghua Lei; Yuqing Zhang. Association of Thigh Intramuscular Fat Infiltration With Incident Knee and Hip Osteoarthritis: A Longitudinal Cohort Study. [Arthritis Rheumatol 2025 Sep]<br>(8) Jie Wei; Chao Zeng; Guanghua Lei; Yuqing Zhang. Gout Flares and Mortality After Sodium-Glucose Cotransporter-2 Inhibitor Treatment for Gout and Type 2 Diabetes. [JAMA Netw Open 2023 Aug]<br>(9) Jie Wei; Chao Zeng; Guanghua Lei; Yuqing Zhang. Postoperative Weight Loss After Antiobesity Medications and Revision Risk After Joint Replacement. [JAMA Netw Open 2025 Feb]<br>(10) Jie Wei; Yilun Wang; Chao Zeng; Guanghua Lei; Yuqing Zhang. Association of a Healthy Lifestyle With All-Cause and Cause-Specific Mortality Among Individuals With Probable Sarcopenia: Population-Based Cohort Study. [JMIR Aging 2025 Jul]<br>(11) Ying Hu; Yilun Wang; Yuqing Zhang; Hongyi He; Chao Zeng; Guanghua Lei; Jie Wei. Genetic susceptibility, walking activity and the risk of incident gout [Rheumatology 2026 Feb]<br>(12) Ying Hu; Hongyi He; Yuqing Zhang; Yilun Wang; Chao Zeng; Guanghua Lei; Jie Wei. Association of walking volume and intensity with incident gout: a population-based cohort study. [American Journal of Epidemiology 2026 Mar]
2. Please explain the key elements of your study design and the use of the available datasets that make your study an original scientific contribution

We conducted a large-scale prospective analysis using the UK Biobank cohort to investigate the associations of purposeful and incidental walking steps with incident falls, number of falls, and incident hip fracture. Our study Our study has several novel features. First, we distinguished walking steps according to cadence-derived intensity by separating purposeful walking steps (⩾40 steps/min) from incidental walking steps (<40 steps/min), rather than focusing solely on total daily step counts. Second, we simultaneously examined multiple clinically relevant fall-related outcomes, including incident falls, number of falls, and incident hip fracture, within a unified analytical framework. Third, we leveraged objectively measured accelerometer-derived walking data, long-term follow-up, and comprehensive adjustment for potential confounders to evaluate the relationship between walking patterns and fall risk. To our knowledge, few studies using the UK Biobank have examined the differential associations of purposeful and incidental walking steps with fall-related outcomes.

3. Please list all publications that addressed similar research questions in the same dataset and indicate where you cited them in your paper

Lloyd L Y Chan, Carmen Herrera Arbona, et.al. Prediction of injurious falls in older adults using digital gait biomarkers extracted from large-scale wrist sensor data. [Age Ageing 2023 Sep]. This paper is cited in the Introduction section as Reference 15.

Shen F, Liu YY, Yang JQ, et.al. Accelerometer-Derived Physical Activity Associated With Incident Probable Sarcopenia, Fracture, and Falls. [Am J Prev Med 2026 Apr]. This paper is cited in the Introduction section as Reference 16.

Luo Y, Jiang Y, Tan S, et.al. Accelerometer-derived physical activity and risk of incident fractures: a population-based cohort study. [Int J Surg 2025 Nov] This paper is cited in the discussion section as Reference 39.

4. Please explain how you addressed multiple testing through an appropriately rigorous statistical threshold and indicate this in the methods section

As our analyses were conducted to test pre-specified hypotheses rather than to perform multiple parallel exploratory comparisons, formal correction for multiple testing was not applied. Statistical significance was defined as a two-sided $P < 0.05$.

5. Please declare to what extent have AI chatbots been used in developing your paper and to which parts of the paper did they contribute

AI chatbots (e.g., ChatGPT) were used solely for minor language editing and grammar refinement. They were not involved in study design, data analysis, interpretation of results, or the generation of scientific content. All scientific content, interpretations, and conclusions were developed by the authors, who reviewed and approved the final manuscript.

Table S2 Baseline characteristics of incidental walking steps categories for incident falls.

Characteristics	Overall	Incidental walking steps				SMD
		Reference (≤2,999)	Second (3,000-3,999)	Third (4,000-4,999)	Fourth (≥5,000)	
Participants, No.	90,932	16,465	31,691	27,706	15,070	
Age, years, mean (SD)	61.79 (7.9)	63.53 (7.7)	62.10 (7.8)	61.23 (7.8)	60.25 (7.8)	0.23
Men, No. (%)	40012 (44.0)	10123 (61.5)	15326 (48.4)	10217 (36.9)	4346 (28.8)	0.38
White, No. (%)	87783 (96.5)	15961 (96.9)	30681 (96.8)	26757 (96.6)	14384 (95.4)	0.05
BMI, kg/m2, mean (SD)	26.69 (4.5)	28.13 (4.9)	27.03 (4.5)	26.15 (4.2)	25.38 (4.0)	0.34
Deprivation index, mean (SD)	-1.74 (2.8)	-1.48 (3.0)	-1.75 (2.8)	-1.85 (2.8)	-1.77 (2.8)	0.07
College, No. (%)	39192 (43.5)	7433 (45.7)	14343 (45.7)	11652 (42.5)	5764 (38.6)	0.08
Alcohol, No. (%)*						0.05
Current	85767 (94.4)	15384 (93.5)	30066 (95.0)	26225 (94.7)	14092 (93.6)	
Never	2627 (2.9)	513 (3.1)	776 (2.5)	783 (2.8)	555 (3.7)	
Previous	2458 (2.7)	554 (3.4)	820 (2.6)	677 (2.4)	407 (2.7)	
Vision problem, No. (%)	6101 (18.8)	1414 (23.9)	2196 (19.2)	1628 (16.7)	863 (16.2)	0.11
BMD, g/cm2, mean (SD)	0.55 (0.1)	0.55 (0.1)	0.55 (0.1)	0.55 (0.1)	0.55 (0.1)	0.04
Fractured/broken bones in last 5 years, No. (%)	7216 (7.9)	1210 (7.3)	2490 (7.9)	2209 (8.0)	1307 (8.7)	0.03
Osteoporosis (%)	883 (1.0)	174 (1.1)	304 (1.0)	237 (0.9)	168 (1.1)	0.02
Dementia No. (%)	31 (0.0)	9 (0.1)	16 (0.1)	2 (0.0)	4 (0.0)	0.02
Osteoarthritis, No. (%)	7216 (7.9)	1389 (8.4)	2532 (8.0)	2150 (7.8)	1145 (7.6)	0.02
Frailty, No. (%)	1654 (1.8)	566 (3.4)	562 (1.8)	352 (1.3)	174 (1.2)	0.08
Health status, No. (%)*						0.19
Excellent	19850 (21.9)	2821 (17.2)	6728 (21.3)	6549 (23.7)	3752 (25.0)	
Good	54534 (60.1)	9305 (56.7)	19125 (60.5)	16928 (61.2)	9176 (61.0)	
Fair	14077 (15.5)	3510 (21.4)	4990 (15.8)	3708 (13.4)	1869 (12.4)	
Poor	2250 (2.5)	768 (4.7)	776 (2.5)	466 (1.7)	240 (1.6)	
Total sleep time, hours/day, mean (SD)	8.42 (1.4)	9.05 (1.7)	8.54 (1.3)	8.23 (1.2)	7.83 (1.2)	0.48
Accelerometer wear time, hours/day, mean (SD)	6.68 (0.6)	6.68 (0.6)	6.69 (0.7)	6.68 (0.6)	6.63 (0.7)	0.05
Purposeful steps, No. (%)						1.06

Reference (≤3,999 steps/d)	11813 (13.0)	6973 (42.4)	4190 (13.2)	627 (2.3)	23 (0.2)
Second (4,000-5,999 steps/d)	24836 (27.3)	5051 (30.7)	11802 (37.2)	6882 (24.8)	1101 (7.3)
Third (6,000-7,999 steps/d)	26786 (29.5)	2625 (15.9)	9120 (28.8)	10520 (38.0)	4521 (30.0)
Fourth (≥8,000 steps/d)	27497 (30.2)	1816 (11.0)	6579 (20.8)	9677 (34.9)	9425 (62.5)

SD – standardized deviation; SMD – standardized mean difference; BMI – body mass index; BMD – bone mineral density.

* Percentage may not sum to100 because of rounding.

Table S3 Baseline characteristics of purposeful walking steps categories for incident hip fracture.

Characteristics	Overall	Purposeful walking steps				SMD
		Reference (≤3,999)	Second (4,000-5,999)	Third (6,000-7,999)	Fourth (≥8,000)	
Participants, No.	94,128	12,398	25,738	27,616	28,376	
Age, years, mean (SD)	61.86 (7.9)	64.80 (7.4)	62.60 (7.9)	61.30 (7.8)	60.45 (7.6)	0.31
Men, No. (%)	41159 (43.7)	6040 (48.7)	11329 (44.0)	11791 (42.7)	11999 (42.3)	0.07
White, No. (%)	90912 (96.6)	11993 (96.7)	24845 (96.5)	26682 (96.6)	27392 (96.5)	0.01
BMI, kg/m2, mean (SD)	26.71 (4.5)	29.29 (5.7)	27.28 (4.5)	26.24 (4.1)	25.52 (3.8)	0.44
Deprivation index, mean (SD)	-1.73 (2.8)	-1.60 (2.9)	-1.86 (2.7)	-1.82 (2.8)	-1.58 (2.9)	0.06
College, No. (%)	40450 (43.4)	4325 (35.4)	10444 (41.0)	12413 (45.4)	13268 (47.2)	0.14
Alcohol, No. (%)*						0.07
Current	88745 (94.4)	11378 (91.9)	24214 (94.2)	26235 (95.1)	26918 (95.0)	
Never	2726 (2.9)	508 (4.1)	787 (3.1)	693 (2.5)	738 (2.6)	
Previous	2572 (2.7)	499 (4.0)	713 (2.8)	669 (2.4)	691 (2.4)	
Smoking, No. (%)*						0.11
Current	6492 (6.9)	1274 (10.3)	1832 (7.1)	1702 (6.2)	1684 (5.9)	
Never	53638 (57.0)	6233 (50.3)	14552 (56.5)	16150 (58.5)	16703 (58.9)	
Previous	33749 (35.9)	4843 (39.1)	9295 (36.1)	9699 (35.1)	9912 (34.9)	
BMD, g/cm2, mean (SD)	0.55 (0.1)	0.54 (0.1)	0.55 (0.1)	0.55 (0.1)	0.55 (0.1)	0.05
Osteoporosis (%)	1062 (1.1)	261 (2.1)	265 (1.0)	266 (1.0)	270 (1.0)	0.05
Diabetes (%), No. (%)	3248 (3.5)	1144 (9.3)	903 (3.5)	668 (2.4)	533 (1.9)	0.18
Osteoarthritis, No. (%)	7698 (8.2)	1778 (14.3)	2326 (9.0)	1932 (7.0)	1662 (5.9)	0.16
Frailty, No. (%)	1776 (1.9)	807 (6.5)	549 (2.1)	255 (0.9)	165 (0.6)	0.19
History of falls; No. (%)						0.10
No falls	77118 (82.1)	9558 (77.2)	21168 (82.3)	23005 (83.4)	23387 (82.6)	
Only one fall	11971 (12.7)	1769 (14.3)	3286 (12.8)	3370 (12.2)	3546 (12.5)	
More than one fall	4898 (5.2)	1046 (8.5)	1265 (4.9)	1201 (4.4)	1386 (4.9)	
Health status, No. (%)*						0.34
Excellent	20450 (21.8)	1453 (11.8)	4930 (19.2)	6529 (23.7)	7538 (26.6)	

Good	56323 (60.0)	6630 (53.7)	15605 (60.8)	16832 (61.1)	17256 (60.9)	
Fair	14727 (15.7)	3289 (26.6)	4437 (17.3)	3792 (13.8)	3209 (11.3)	
Poor	2402 (2.6)	980 (7.9)	685 (2.7)	412 (1.5)	325 (1.1)	
Serum vitamin D level (nmol/L)	49.73 (20.7)	45.95 (20.7)	49.03 (20.7)	50.33 (20.7)	51.39 (20.6)	0.14
Serum calcium level (mmol/L)	2.38 (0.1)	2.38 (0.1)	2.38 (0.1)	2.38 (0.1)	2.38 (0.1)	0.03
Vitamin D supplementation, No. (%)	3975 (4.2)	568 (4.6)	1099 (4.3)	1115 (4.0)	1193 (4.2)	0.01
Calcium supplementation, No. (%)	7001 (7.4)	925 (7.5)	1926 (7.5)	2034 (7.4)	2116 (7.5)	<0.01
Total sleep time, hours/day, mean (SD)	8.42 (1.4)	8.92 (1.7)	8.57 (1.4)	8.37 (1.3)	8.13 (1.3)	0.30
Accelerometer wear time, hours/day, mean (SD)	6.68 (0.6)	6.67 (0.6)	6.69 (0.6)	6.69 (0.6)	6.66 (0.7)	0.03
Incidental steps, No. (%)*						1.09
Reference (≤2,999 steps/d)	17149 (18.2)	7345 (59.2)	5231 (20.3)	2712 (9.8)	1861 (6.6)	
Second (3,000-3,999 steps/d)	32752 (34.8)	4364 (35.2)	12207 (47.4)	9401 (34.0)	6780 (23.9)	
Third (4,000-4,999 steps/d)	28633 (30.4)	663 (5.3)	7149 (27.8)	10832 (39.2)	9989 (35.2)	
Fourth (≥5,000 steps/d)	15594 (16.6)	26 (0.2)	1151 (4.5)	4671 (16.9)	9746 (34.3)	

SD – standardized deviation; SMD – standardized mean difference; BMI – body mass index; BMD – bone mineral density.

* Percentage may not sum to100 because of rounding.

Table S4 Baseline characteristics of incidental walking steps categories for incident hip fracture.

Characteristics	Overall	Incidental walking steps				SMD
		Reference (≤2,999)	Second (3,000-3,999)	Third (4,000-4,999)	Fourth (≥5,000)	
Participants, No.	94,128	17,149	32,752	28,633	15,594	
Age, years, mean (SD)	61.86 (7.85)	63.58 (7.68)	62.18 (7.80)	61.31 (7.82)	60.33 (7.80)	0.23
Men, No. (%)	41159 (43.7)	10475 (61.1)	15719 (48.0)	10495 (36.7)	4470 (28.7)	0.38
White, No. (%)	90912 (96.6)	16630 (97.0)	31725 (96.9)	27660 (96.6)	14897 (95.5)	0.05
BMI, kg/m2, mean (SD)	26.71 (4.53)	28.17 (4.97)	27.05 (4.53)	26.17 (4.21)	25.38 (4.02)	0.34
Deprivation index, mean (SD)	-1.73 (2.82)	-1.47 (2.98)	-1.75 (2.81)	-1.85 (2.75)	-1.77 (2.75)	0.07
College, No. (%)	40450 (43.4)	7688 (45.4)	14765 (45.5)	12042 (42.5)	5955 (38.5)	0.08
Alcohol, No. (%)*						0.05
Current	88745 (94.4)	16006 (93.4)	31073 (95.0)	27082 (94.7)	14584 (93.6)	
Never	2726 (2.9)	537 (3.1)	798 (2.4)	821 (2.9)	570 (3.7)	
Previous	2572 (2.7)	591 (3.4)	851 (2.6)	706 (2.5)	424 (2.7)	
Smoking, No. (%)*						0.07
Current	6492 (6.9)	1495 (8.7)	2270 (6.9)	1761 (6.2)	966 (6.2)	
Never	53638 (57.0)	9286 (54.1)	18550 (56.6)	16540 (57.8)	9262 (59.4)	
Previous	33749 (35.9)	6314 (36.8)	11854 (36.2)	10261 (35.8)	5320 (34.1)	
BMD, g/cm2, mean (SD)	0.55 (0.13)	0.55 (0.14)	0.55 (0.13)	0.55 (0.12)	0.54 (0.13)	0.04
Osteoporosis (%)	1062 (1.1)	207 (1.2)	358 (1.1)	300 (1.0)	197 (1.3)	0.01
Diabetes (%), No. (%)	3248 (3.5)	1135 (6.6)	1162 (3.6)	681 (2.4)	270 (1.7)	0.14
Osteoarthritis, No. (%)	7698 (8.2)	1510 (8.8)	2689 (8.2)	2284 (8.0)	1215 (7.8)	0.02
Frailty, No. (%)	1776 (1.9)	631 (3.7)	590 (1.8)	373 (1.3)	182 (1.2)	0.09
History of falls; No. (%)*						0.03
No falls	77118 (82.1)	13975 (81.6)	27043 (82.7)	23477 (82.1)	12623 (81.1)	
Only one fall	11971 (12.7)	2140 (12.5)	4100 (12.5)	3639 (12.7)	2092 (13.4)	
More than one fall	4898 (5.2)	1005 (5.9)	1571 (4.8)	1475 (5.2)	847 (5.4)	
Health status, No. (%)*						0.19
Excellent	20450 (21.8)	2910 (17.0)	6923 (21.2)	6759 (23.7)	3858 (24.8)	

Good	56323 (60.0)	9630 (56.4)	19748 (60.4)	17449 (61.1)	9496 (61.0)	
Fair	14727 (15.7)	3707 (21.7)	5190 (15.9)	3876 (13.6)	1954 (12.6)	
Poor	2402 (2.6)	839 (4.9)	819 (2.5)	493 (1.7)	251 (1.6)	
Serum vitamin D level (nmol/L)	49.73 (20.73)	46.51 (20.53)	49.56 (20.64)	50.61 (20.57)	51.96 (21.01)	0.14
Serum calcium level (mmol/L)	2.38 (0.09)	2.38 (0.09)	2.38 (0.09)	2.38 (0.09)	2.38 (0.09)	0.02
Vitamin D supplementation, No. (%)	3975 (4.2)	658 (3.8)	1266 (3.9)	1288 (4.5)	763 (4.9)	0.03
Calcium supplementation, No. (%)	7001 (7.4)	993 (5.8)	2134 (6.5)	2366 (8.3)	1508 (9.7)	0.08
Total sleep time, hours/day, mean (SD)	8.42 (1.41)	9.06 (1.67)	8.54 (1.34)	8.23 (1.23)	7.83 (1.22)	0.48
Accelerometer wear time, hours/day, mean (SD)	6.68 (0.61)	6.68 (0.61)	6.69 (0.59)	6.68 (0.61)	6.63 (0.67)	0.05
Purposeful steps, No. (%)*						1.07
Reference (≤3,999 steps/d)	12398 (13.2)	7345 (42.8)	4364 (13.3)	663 (2.3)	26 (0.2)	
Second (4,000-5,999 steps/d)	25738 (27.3)	5231 (30.5)	12207 (37.3)	7149 (25.0)	1151 (7.4)	
Third (6,000-7,999 steps/d)	27616 (29.3)	2712 (15.8)	9401 (28.7)	10832 (37.8)	4671 (30.0)	
Fourth (≥8,000 steps/d)	28376 (30.1)	1861 (10.9)	6780 (20.7)	9989 (34.9)	9746 (62.5)	

SD – standardized deviation; SMD – standardized mean difference; BMI – body mass index; BMD – bone mineral density.

* Percentage may not sum to100 because of rounding.

Table S5 Association between the walking steps and the risk of incident hip fracture.

Purposeful walking steps	Reference (<3,999)	Second (4,000-5,999)	Third (6,000-7,999)	Fourth (≥8,000)
No.	12398	25738	27616	28376
Incident case, No.	117	131	120	117
Incident rate (per 1000 person-years)	1.39	0.73	0.62	0.59
Follow-up time, year (mean)	6.77	6.94	6.99	7.01
Rate difference (95% CI)		-0.66 (-0.94, -0.38)	-0.77 (-1.05, -0.49)	-0.80 (-1.07, -0.53)
HR (95% CI)				
Crude model	1.00 (reference)	0.65 (0.51, 0.84)	0.63 (0.48, 0.81)	0.66 (0.51, 0.86)
Model 1*	1.00 (reference)	0.56 (0.35, 0.90)	0.44 (0.27, 0.73)	0.34 (0.20, 0.58)
Model 2†	1.00 (reference)	0.63 (0.39, 1.02)	0.49 (0.29, 0.83)	0.36 (0.21, 0.62)
**Incidental walking steps**	**Reference (≤2,999)**	**Second (3,000-3,999)**	**Third (4,000-4,999)**	**Fourth (≥5,000)**
No.	17149	32752	28633	15594
Incident case, No.	117	150	127	91
Incident rate (per 1000 person-years)	1.00	0.66	0.63	0.83
Follow-up time, year (mean)	6.83	6.95	6.99	7.01
Rate difference (95% CI)		-0.34 (-0.55, -0.13)	-0.37 (-0.58, -0.16)	-0.17 (-0.42, 0.08)
HR (95% CI)				
Crude model	1.00 (reference)	0.77 (0.60, 0.98)	0.81 (0.63, 1.04)	1.17 (0.89, 1.54)
Model 1*	1.00 (reference)	0.57 (0.36, 0.90)	0.51 (0.31, 0.84)	0.59 (0.32, 1.07)
Model 2‡	1.00 (reference)	0.70 (0.44, 1.11)	0.73 (0.43, 1.24)	0.98 (0.51, 1.85)

RD – rate difference; HR – hazard ratio; CI – confidence interval.

* Model 1 is adjusted for age, sex, ethnicity, education level, deprivation, alcohol consumption status, smoking status, health status, accelerometer wear time, BMI, daily sleep time, frailty, BMD, osteoporosis, osteoarthritis, diabetes, history of falls, serum vitamin D level, serum calcium level, vitamin D supplementation, and calcium supplementation.

† Model 2 is adjusted for Model 1 variables plus incidental walking steps.

‡ Model 2 is adjusted for Model 1 variables plus purposeful walking steps.

References

1 Hanlon P, Nicholl BI, Jani BD, Lee D, McQueenie R, Mair FS. Frailty and pre-frailty in middle-aged and older adults and its association with multimorbidity and mortality: a prospective analysis of 493 737 UK Biobank participants. Lancet Public Health. 2018;3:e323-e32.

2 Fried LP, Tangen CM, Walston J, Newman AB, Hirsch C, Gottdiener J, et al. Frailty in older adults: evidence for a phenotype. J Gerontol A Biol Sci Med Sci. 2001;56:M146-56.
